# Public health research needs for molecular epidemiology and to emphasize homeostasis – could the omnipotent endopeptidase inhibitor α-2-macroglobulin be a meaningful biomarker?

**DOI:** 10.12688/f1000research.19781.1

**Published:** 2019-07-09

**Authors:** Frank Peter Schelp, Ratthaphol Kraiklang, Benja Muktabhant, Pornpimon Chupanit, Pattara Sanchaisuriya

**Affiliations:** 1 Faculty of Public Health, Khon Kaen University, Khon Kaen, 40002, Thailand

**Keywords:** Public health, non-communicable diseases, sustainable development goals, biomarkers, dietary restriction, homeostasis, metabolic syndrome, alpha-2-macroglobulin

## Abstract

Public health authorities in low- and middle-income countries face dramatic challenges in handling rapidly increasing non-communicable diseases (NCDs), due to the epidemiological- and particularly nutritional transition. Among major reasons for the development of NCDs are smoking and alcohol, but overnutrition and obesity are also major threats to population health. Obesity is related to diabetes and cancer, but also has a genetic background. It is difficult to recommend a healthy nutrition. This is because of conflicting nutritional conceptions, and given the complexity of human metabolism understanding this topic can be difficult for the laymen.  Public health measures advocating physical activity and refraining from high intake of energy, sugar and soft drinks need to be enhanced by supporting the ‘intrinsic motivation’ to preserve a good health. The mission of public health should be to increase awareness about the complexity of human metabolism, and the involvement of genetic and epigenetics in health and diseases. To maintain homeostasis, means to keep an optimal relationship between catabolism and synthesis, seems to be of particular interest. Preconditions for this is, that public health institutions within the administration- and academic sector follow up developments in life science and molecular biology and conduct population-based research making use of molecular epidemiology, especially those related to key metabolic steps and maintenance of ‘homeostasis’, in balancing catabolism and anabolism. A prospective biomarker for this situation might be α-2-macroglobulin.

## Background

Non-communicable diseases (NCDs), such as cardiovascular diseases, diabetes, chronic obstructive pulmonary diseases and cancer, are the main focus of the Sustainable Development Goals (SDGs) ^1^. Target 3.4 of the SDGs intends to decrease premature death through NCDs by one third up to 2030. The four main risk factors mentioned are smoking, ‘unhealthy’ diets, physical inactivity and ‘harmful’ use of alcohol. Governments and public health authorities are encouraged to enforce so called 16 ‘best buy’ strategies to reach the target. While it is well accepted that tobacco smoking and alcohol abuse are harmful for health and there is general agreement not to smoke and at least to refrain from too much or excessive alcohol consumption, campaigning against ‘unhealthy’ diets is more problematic. This is because of the complexity of how nutrition relates to health. The ‘global obesity pandemic’ ^2^ not only is caused by a surplus of energy in the diet, but it’s link to diabetes ^3^ and cancer ^4, 5^ and other diseases is driven by complex metabolic pathways ^6– 9^. Not only does overnutrition play a role in the development of cardiovascular diseases, cancer, diabetes and influences aging, but the risk for obesity is also related to genetic factors ^10, 11^. Through epigenetic pathways under- and overnutrition of pregnant females might result in diabetes, cancer and cardiovascular diseases in their adult children ^12– 15^. Within the field of nutritional sciences matters are further complicated by recent developments of contradictory nutritional conceptions. There seems to be disagreement about what a healthy and appropriate diet should be and controversial opinions are justified by supportive investigations into molecular factors from both sides when either a ‘low fat high carbohydrate-‘ or a ‘low-carbohydrate, high fat diet’ are promoted ^16^.

## The role of molecular epidemiology in public health actions against overnutrition

Governmental authorities and public health institution, overseeing the wellbeing of the population, cannot capitulate and stop promoting ‘healthy’ nutrition, in view of the constraints. So far major public health tools for working against NCDs, in regard to nutrition, are encouragement of physical activity, trying to influence behavior and practice through sophisticated methods of social sciences ^17^, and to increase taxes on harmful products such as sugar and soft drinks with the intention to reduce consumption ^18^. Benefit and drawbacks of these methods are not questioned here, but it has been argued by Slattery (2002) that ‘while working at the population level of exploration, molecular epidemiology must incorporate knowledge from many disciplines to obtain an understanding of the organism, the system and the cell. Translating complex disease pathways into relevant public health messages should be the goal and the result of the art of epidemiology’ ^19^. Far too often interesting results within the field of genetics, insight into metabolic pathways and molecular components, such as enzymes and cytokines, escape the intention of those working in public health. This is plausible, as investigations using laboratory models, worms and rodents, are by no means research tools for public health. Studies exploring epigenetic effects, such as DNA methylation, as recently conducted in Estonia ^20^ are most probably not feasible for large scale epidemiological studies in low- but also middle- and high middle income countries. However, low- and middle-income countries need to make use of advances in public health. In particular low-, but especially high middle-, income countries should have the means to follow up developments in molecular epidemiology in order to have a deeper understanding of the nature of NCDs, and should conduct as much population-based research as possible, including the use of promising biomarkers to give an insight into genetic and metabolic pathways.

In fact, besides anthropometry measurements, a number of clinical laboratory methods and biomarkers are already in common use and in future, epigenetic and molecular epidemiology could be additional suitable aspirants for population-based studies ^19, 21, 22^. However, multiple constraints in the use of biomarkers, as outlined for metabolic syndrome (MetS) should be considered.

## Pros and cons of biomarkers – the examples of the components of the metabolic syndrome

In assessing the nutritional status using the body mass index (BMI) and other measurements as independent variables, MetS is frequently used as dependent variable since it incorporates a number of factors related directly or indirectly to NCDs. Variables associated with the syndrome include elevated blood glucose, dyslipidemia, abdominal obesity and high blood pressure. There are five different definitions of MetS, with different thresholds of its components. Because of this ,and other controversial arguments, attempts have failed to agree on either version ^23^. As a compromise the use of the so called ‘harmonized version’ of MetS has been recommended ^24^. It is now considered that study participants categorized as exposed to MetS should be selected if they display three or more of the five criteria. However, this results in arbitrary groupings of individuals belonging to the ‘MetS group’, and individuals are integrated with one or two factors of MetS but less than three into the ‘non-MetS’ group. An example of using the ‘harmonized version’ MetS version in grouping study participants into the MetS- and the non-MetS group is given in Table 2 of a recent publication ^25^. To apply the ‘harmonized version’ weakens the validity of MetS as a dependent variable. The ‘nature’ of MetS as a ‘syndrome’ is also questioned. The term ‘syndrome’ should be used in case ‘the whole is greater than the parts’ ^26^, but this is doubtful ^27^ and before selecting MetS as independent variable it should be considered that a number of factors influence MetS such as age, sex and ethnicity.

## Biomarkers representing key factor for the metabolism

As mentioned above, the use of MetS seems to be problematic and the recommendation of ‘waist circumference’ as a strong indicator of obesity and ‘insulin resistance’ as one of the metabolic key factors, could be a worthwhile alternatives for MetS ^24^. Waste circumference is a good measurement for overnutrition, because energy intake in excess is stored in the abdominal fat tissue. The adipokines of the fat tissue, by excreting inflammatory molecules, increase the risk to develop diabetes and cancer. Insulin resistance is ‘the intersection’ either for the way to health or to metabolic disturbances. The absence of a general standard for waist circumference, however, is a disadvantage, and waist circumference needs to be standardized for different population groups ^28^, but with the homeostatic model assessment (HOMA) on hand, a method is available for estimating insulin resistance in epidemiological studies ^29^.

Trying to find and test biomarkers mirroring key metabolic steps for health and disease should be one major objective for molecular epidemiology. It is equally important to gain insight into the interaction of anabolism with catabolism. A candidate for the latter aspect is α-2-macroglobulin (α2M). The importance of α2M has been mentioned by Ohlsson 1972, stating that ‘α2M’ may have a key role in the body’s protection against autodigestion (cited by Schelp FP *et al.* ^30^), which implies that a complete deficiency will not be compatible with life. Within human plasma α2M is the largest non-immunoglobulin, and an almost omnipotent inhibitor of endopeptidases with a unique way to deactivate proteinases ^31^. The inhibitor is found in all mammals, and the biological significance for growth and differentiation can be judged by its presence during embryogenesis, pregnancy, childhood and in aging ^32, 33^. A comprehensive overview about the molecular structure of α2M, mechanism of action, function and pathophysiology is given in the review article from Rehman *et al.* (2013) ^34^.

## α-2-Macroglobulin in health and disease

Besides the attention molecular biology has given to α2M, in clinical settings the inhibitor was found to be related to the development of Alzheimer’s ^35^. Low α2M levels were observed in some patients with lung diseases ^36^, and in advanced prostate cancer ^37^, while in breast- ^38^ and bladder cancer ^39^ elevated levels of α2M were observed. It has been hypothesized that induced increase of endogenous proteinase inhibitors is protective against cancer ^40^. The assumption, among others, was based on the ‘fat-related-cancer’ hypothesis ^41, 42^, and the low risk of vegetarians for cardiovascular diseases and cancer ^43^, as well as the finding that α2M concentrations and other proteinase inhibitors were higher in Thai vegetarians compared with omnivores ^44^. It was argued that the balance between proteinases and their inhibitors are regulators of tumor growth. The role of proteinase inhibitors in connection with cancer protection considered a number of different inhibitors, and the specific role of α2M remains vague since α2M is incorporated in normal but not in tumor cells. In laboratory mice alpha macroglobulin is active through ‘biomediators’ but not through its inhibitory capacity ^45^. So far it was concluded that α2M has a role in controlling normal but not malignant growth ^46, 47^.

## The role of α-2-macroglobulin in maintaining homeostasis in ‘dietary restriction’

Obesity, as outlined above, is detrimental for health but ‘dietary restriction’, defined as ‘reduced food intake by avoiding malnutrition’, extends life span in animals and humans ^48– 50^. It has been demonstrated recently, by using a mouse model, that epigenetic modification in dietary restriction delayed aging and changed the gene expressions of the lipid profile ^51^. ‘Subclinical undernutrition’ in preschool children could reflect ‘dietary restriction without malnutrition’. The two forms of the condition are ‘wasting’, a deficit in weight for height and ‘stunting’, a deficit in height for age, adjusted to a standard ^52^. Children categorized as wasting and stunting are apparently healthy without clinical signs of undernutrition. A number of investigation in Bangkok and villages in rural Thailand disclosed elevated α2M levels and low 3-methylhistidine (3MH) urine excretion in healthy, age matched, village children in comparison to their Bangkok counterparts ^53^. 3MH is supposed to reflect muscle breakdown ^54, 55^. A similar result was obtained when comparing normal nourished preschool children with those deficient in weight for height. α2M serum concentration in the marginal nourished children increased over their well-nourished village counterparts ^56^. In an animal experiment with laboratory growing rats under a marginal diet, with altered protein and energy content, serum proteinase inhibitors increased and 3HM decreased ^57^. The results of the investigations support the hypothesis that in the situation of ‘dietary restriction’ proteinase inhibitors, including α2M, decrease muscle catabolism, and in the case of the village children kept them healthy though ‘an optimal relationship between catabolism and synthesis, thus resulting in stunting’ ^58^. In case ‘marginal nutritional intake’, results in elevated α2M serum concentrations, as expected, overweight and obese Thai adults in Bangkok, showed lower α2M serum levels compared with normal individuals. The proteinase inhibitor selected as dependent variables in a multiple regression, resulted in a model including age, sex HDL cholesterol and BMI ^59^. A similar result was obtained studying hard working male construction laborers, in that a negative correlation were found for the variables age, weight, height, BMI, arm- and midarm circumference, triceps skinfold and HDL with α2M as the dependent variable ^60^. α2M of female construction workers did relate to any of the variables investigated. A dietary survey conducted with apparently health Thai farmers found a statistically significant negative correlation of α2M with energy, protein, fat and carbohydrate intake ^61^. All the results obtained from the variety of different studies seem to be in accordance with the assumption that α2M supports homeostasis in situations of a ‘challenged’ nutritional status.

## α-2-Macroglobulin in protein-energy-malnutrition

All the results obtained from the variety of different studies, as reviewed here, seem to be in accordance with the notion that α2M supports homeostasis in situations of a ‘challenged’ nutritional status and suggest that proteinase inhibitors play a key role in maintaining the metabolism in balance. This also was assumed in observing patients suffering from clinical protein-energy malnutrition (PEM). The situation in PEM children is different from subclinical malnutrition. While increased proteinase inhibitors in wasting and stunting children somehow delay catabolism to maintain homeostasis, proteinase inhibitors increasing in seriously malnourished children, interrupt the mobilization of endogenous proteins which are needed to maintain homeostasis, for instance by providing essential amino acids. The increase of proteinase inhibitors aimed to counteract the proteases released in the course of infection, turn marasmus patients to develop clinical symptoms of kwashiorkor ^30^. Comparing marasmus with kwashiorkor, the latter is the more serious condition.

## Different levels of homeostasis

‘Homeostasis’, as a metabolic condition depends on age, sex, genetic and environmental factors, and is maintained on different levels. This has been pointed out by Pontzer (2015) using the example of energy expenditure and energy balance ^62, 63^. This message from an evolutionary anthropologist is particularly important for public health in relation to physical activity. It is known that to lose weight by exercising has its limits. Increasing physical activity will lead to weight loss, however, excessive physical activity will not increase weight loss with increased energy expenditure; instead energy is acquired by using available resources used for basic functions of the organism under normal conditions. The consequence of this can be deadly in untrained individuals partaking in extreme physical activity e.g. a long run over 40 km. Winners of international marathon events are usually of African descent, whose ‘homeostasis’ allows them to cover the marathon distance in about two hours, while the rest of the field, using the rest of the day to finish the run. Homeostasis obviously can be achieved on different levels. The metabolism of the marathon winner allows them to economize energy expenditure much more efficiently as compared to other participants. In the regulation of the delicate balance of efficient energy expenditure, α2M might play a key role and might help to better understand the mechanisms regulating total energy expenditure. Recently a genetic hint towards regulating endurance and fatigue of muscles has been described in a rodent model ^64^. Research in this direction might be a further step to allow a better understanding of important metabolic pathways related to energy expenditure and endurance.

Other important issues for public health are also waiting for further exploration, such as how to distinguish biological- from chronological age ^65, 66^ and whether ‘dietary restriction’ is ‘beneficial’ for health and if so, are there drawbacks to be observed under certain circumstances and different ages. So for instance higher α2M levels in connection with some biological substances called ‘metallothioneins’ are beneficial in young adults but might have a harmful role in aging ^32^.

## Conclusion

In view of the challenge of non-communicable diseases, the aim of those caring for the health of the population should support the ‘intrinsic motivation’ of individuals to remain healthy. This might not only be achieved by encouraging physical activity, and restrain from smoking, alcohol and overeating. Motivation to change ‘bad’ behavior and maintaining ‘good behavior’, requires understanding what ‘bad’ behavior is meant to be, and what ‘homeostasis’ means and how to maintain it. The nature of NCDs are not yet well understood. This hampers the formulation of clear recommendations for ‘healthy’ behavior and limits the trust of the general public in health messages. Public health authorities at least should try to follow up developments and assess the significance of what is increasingly becoming known about our metabolism. Last but not least, public health should contribute to translate findings in life science, by conducting research applying molecular epidemiology, in such a way that these findings are relevant for human population groups, and may even validate α2M as a meaningful biomarker.

## Data availability

### Underlying data

No data are associated with this article.
