# Supplementary material for: Public health research needs for molecular epidemiology and to emphasize homeostasis – could the omnipotent endopeptidase inhibitor α-2-macroglobulin be a meaningful biomarker?
Source: F1000Res. 2019 Jul 9;8:1025. [Version 1] doi: 10.12688/f1000research.19781.1 (PMC6880254; doi:10.12688/f1000research.19781.1)
Supplement: Peer review history

# Peer review: Reviewer response for version 1

**DOI:** 10.5256/f1000research.21700.r53054

**Published:** 2019-11-21

**Authors:** Chaowanee Chupeerach

**Affiliations:** 1 Institute of Nutrition, Mahidol University, Nakhon Pathom, Thailand

The manuscript is a good explanation of the need of molecular epidemiology in the public health area. This article reported the role of alpha 2 macroglobulin in the difference of metabolic state and it might be associated to homeostasis and health status. Therefore it could be a public health concern in the future. 

 However, the abstract should re-arranged to be more representative of the manuscript summary.

 For the review body, to interpret and follow the role of alpha 2 macroglobulin, the reader might need more information about this protein in terms of molecular detail.

I confirm that I have read this submission and believe that I have an appropriate level of expertise to confirm that it is of an acceptable scientific standard.

# Peer review: Reviewer response for version 1

**DOI:** 10.5256/f1000research.21700.r51079

**Published:** 2019-09-03

**Authors:** Florian J. Schweigert

**Affiliations:** 1 Department of Physiology and Pathophysiology of Nutrition, University of Potsdam, Potsdam, Germany

The submitted manuscript is a very well written review on the possible use of a2-macroglobulin as a marker of metabolism. The manuscript describes the general need of valid and meaningful biomarkers in the context of NCDs in public health research, especially with regard to overnutrition and obesity. The manuscript covers the implication of overnutrition and obesity for public health and the need of biomarkers and suggests a2-macroglobulin as such. The main part of the manuscript describes in very much detail the current knowledge of a2M in metabolic function as an omnipotent inhibitor of endopeptidases. With regard to the topic of the manuscript the authors concentrate on the knowledge regarding a2M in energy homeostasis especially with regard to energy-protein malnutrition.

 Comments:

- In general the manuscript itself is well written and balanced. However, the abstract is by no means reflecting the manuscript. It lacks condensed information on a2M and only addresses the general aspect of public health and the need of a biomarker.
- It is challenging for the reader to cite a citation with refers to another citation. It cannot be retrieved without greater efforts. See second section, page 4 of Ohlsson (1972), as cited by Schelp *et al.*

I confirm that I have read this submission and believe that I have an appropriate level of expertise to confirm that it is of an acceptable scientific standard.
